# Sources of Polyphenols with Potential α-Glucosidase-Inhibitory Activities from Thai Local Edible Plants: In Vitro and Network Pharmacology Approaches

**DOI:** 10.3390/plants15111721

**Published:** 2026-06-02

**Authors:** Ploenthip Puthongking, Juthamat Ratha, Pimolwan Siriparu, Muhammad Subhan, Panyada Panyatip, Ployvadee Sripadung, Tanit Padumanonda, Sarin Tadtong, Vuanghao Lim, Bunleu Sungthong

**Affiliations:** 1 Division of Pharmaceutical Chemistry, Faculty of Pharmaceutical Sciences, Khon Kaen University, Khon Kaen 40002, Thailand; pploenthip@kku.ac.th; 2 Melatonin Research Group, Khon Kaen University, Khon Kaen 40002, Thailand; juthra@kku.ac.th; 3 Graduate School, Faculty of Pharmaceutical Sciences, Khon Kaen University, Khon Kaen 40002, Thailand; pimolwan.s@kkumail.com (P.S.); muhammad.s@kkumail.com (M.S.); 4 Department of Pharmacognosy, Faculty of Pharmacy, Srinakharinwirot University, Nakhon Nayok 26120, Thailand; panyada@g.swu.ac.th (P.P.); sarin@g.swu.ac.th (S.T.); 5 Integrative Pharmaceuticals and Innovation of Pharmaceutical Technology Research Unit, Faculty of Pharmacy, Mahasarakham University, Maha Sarakham 44150, Thailand; ployvadee.s@msu.ac.th; 6 Division of Pharmacognosy and Toxicology, Faculty of Pharmaceutical Sciences, Khon Kaen University, Khon Kaen 40002, Thailand; tanpad@kku.ac.th; 7 Pusat Kanser Tun Abdullah Ahmad Badawi, Universiti Sains Malaysia, Bertam, Kepala Batas 13200, Penang, Malaysia; vlim@usm.my

**Keywords:** phenolic acids, tryptophan, melatonin, edible plants, diabetes mellitus (DM)

## Abstract

Plant polyphenols possess various biological activities such as antioxidant and antidiabetic activities. Based on the high plant biodiversity in Thailand, selected edible plants were therefore investigated to explore how dietary polyphenols and α-glucosidase-inhibitory activity can contribute to significant control and prevention of type 2 diabetes mellitus (T2DM). Network pharmacology of antidiabetic activity was employed to predict the underlying antidiabetic mechanisms of the phytochemicals identified in the 15 selected edible Thai plants. The results showed that wild guava (*Careya arborea* Roxb.; CaA) leaf extract presented the highest total phenolic content (TPC). CaA exhibited the highest antioxidant activities in all the assays as a function of its TPC. Moreover, CaA showed the highest inhibitory effect on α-glucosidase activity in the test, with an IC_50_ of 0.13 µg/mL, being approximately 1600 times more potent than the standard α-glucosidase-inhibitory drug acarbose. Phenolic profile analysis revealed that the CaA leaf extract consisted of gallic acid, caffeic acid, sinapic acid, rutin, and quercetin. The phytochemical contents of CaA strongly contributed to its antioxidant and α-glucosidase-inhibitory activity. In addition to other phytochemicals, the highest contents of the indolamine compounds tryptophan and melatonin were found in malabar spinach (*Basella alba* L.; BA) and water clover (*Marsilea crenata* C. Presl; MaC), at 2213.05 and 99.17 ng/g DW, respectively. Network pharmacology on antidiabetic activity exhibited the most relevant pathway involved in the insulin resistance and AGE-RAGE signaling pathways that caused glucose and glycogen synthesis in skeletal muscle, increasing hepatic gluconeogenesis, and reduced glycogen synthesis in the liver. The implication of these findings is that these Thai edible plants have the potential to combat diabetes by inhibiting the function of α-glucosidase.

## 1. Introduction

Diabetes mellitus (DM) is one of the most common chronic diseases globally, characterized by abnormally high levels of glucose in the bloodstream, which is called hyperglycemia. An epidemiological study by Guariguata et al. [1] projected that the prevalence of DM will increase from 382 million people in the year 2013 to 592 million by 2053. Inhibiting α-glucosidase is one of the targets for treating DM. Many plants have been reported as α-glucosidase inhibitors [2], and a number of edible plants have been proposed to be useful in the treatment of diabetes. Edible plants are promising sources of compounds with human health benefits and medicinal properties, including important nutrients (essential proteins, dietary fiber, vitamins, and minerals) and bioactive compounds, especially polyphenols [3].

The prevalence of diabetes has significantly increased to about 9.6% of the total Thai adult population [4]. One therapeutic approach in treating type 2 diabetes mellitus (T2DM) is retarding the absorption of glucose by inhibiting digestive enzymes, such as α-glucosidase, thereby slowing the rate of carbohydrate digestion and glucose absorption in the small intestine [5]. This has proved to be one of the best strategies for decreasing postprandial blood glucose and delaying diabetic complications. Previous studies have reported a direct relationship between phenolics or flavonoids in plant extracts and α-glucosidase-inhibitory activity [6,7]. The high diversity of Thai edible plants, particularly in the northeastern region, offers a vast resource for antidiabetic agents [2,8].

Polyphenols are a large class of phytochemical compounds widely distributed across plants, which produce them as secondary metabolites; they include phenolic acids, flavonoids, stilbenes, coumarins, and indolamines [9,10]. Polyphenols are commonly found as the main natural antioxidants associated with health benefits [11,12]. HPLC techniques are typically applied to qualitatively and quantitatively analyze these polyphenol phytochemicals [8,9,13,14]. The consumption of dietary polyphenols has been reported to reduce the risk of chronic diseases such as cardiovascular, diabetic, and neurological disorders [10].

Melatonin is an endogenous molecule produced in plant species. Its biosynthesis pathway involves four enzymatic steps and two important precursors including tryptophan [15]. Melatonin is also well-known as a regulator of the circadian rhythm, mainly released from the pineal gland in animals. Melatonin directly influences both glucose homeostasis and insulin secretion from β-cells, which regulate glucose metabolism [16,17]. Circadian disruption is defined as misalignment between the endogenous circadian system and behavioral cycles. Misalignment between components of the circadian rhythm and daily rhythms such as sleep–wake behavior or food intake, genetic inheritance, and environmental or behavioral factors might contribute to insulin resistance [18]. The disruption of melatonin in the circadian rhythm could therefore result in poor glycemic control [16].

As part of our efforts to explore dietary polyphenols and α-glucosidase-inhibitory activity in 15 Thai local edible plants, we found strong evidence from a previous report that dietary polyphenols can help to control and prevent type 2 diabetes mellitus (T2DM) [19]. Although several edible plants in Thailand have been reported for their antidiabetic properties [8], research on the relationships between phytochemical compounds and such activities of selected plants from northeastern Thailand has been limited. This study therefore aimed to identify and qualify phenolic and indolamine compounds using HPLC analysis. The antioxidant and α-glucosidase-inhibitory activities of 15 edible plants from northeastern Thailand were also evaluated. Principal component analysis (PCA) and clustering analysis were carried out to predict the phytochemical contents to which antioxidant and α-glucosidase-inhibitory activities could be attributed. Network pharmacology was also conducted to predict the potential therapeutic mechanisms of polyphenols in the treatment of T2DM.

## 2. Results and Discussion

The selected plants have been reported as the most popular side dishes, especially in the Northeast of Thailand. Even though some previous research has indicated the biological properties of these plants, our study focused on different aspects. Therefore, 15 northeastern Thai edible plants were selected to comparatively analyze their contents of tryptophan, melatonin, total phenolic compounds, and total flavonoids; phytochemical profiles; and biological activities such as antioxidant and α-glucosidase-inhibitory activities.

### 2.1. Phytochemical Investigation

#### 2.1.1. HPLC-FD Analysis of Tryptophan and Melatonin Contents

The contents of serotonin, tryptophan, and melatonin in the 15 plants were determined. Tryptophan and melatonin were found in 6 and 3 of the plant extracts, respectively, but no serotonin was detected in any (Table 1). Tryptophan is an essential amino acid and also a precursor for melatonin synthesis. The content of tryptophan ranged from 15.75 to 2213.05 ng/g dry weight (DW). The highest content of tryptophan was obtained from Malabar spinach (*Basella alba* L.; BA), followed by red lucky seed (*Adenanthera pavonine* L.; AP), water clover (*Marsilea crenata* C. Presl; MaC), Vietnamese coriander (*Persicaria odorata* (Lour.) Soják; PO), bitter melon (*Momordica charantia* (L.); MoC), and Hill glory bower (*Clerodendrum infortunatum* L.; CI), respectively. The melatonin contents were the highest in MaC (99.17 ng/g DW), followed by Fish mist (*Houttuynia cordata* Thunb.; HC) (4.38 ng/g DW) and MoC (2.44 ng/g DW).

#### 2.1.2. Total Phenolic and Total Flavonoid Contents

The total phenolic contents (TPCs) in 15 Thai local edible plant extracts ranged from 9.36 to 606.77 mg GAE/g DW as shown in Table 1. The highest TPC was found in wild guava (*Careya arborea* Roxb.; CaA) extract, while Chinese cabbage (*Brassica rapa* L.; BR) extract had the lowest TPC (sixty-five times lower than CaA extract). The total flavonoid contents (TFCs) of all 15 extracts ranged from 2.11 to 16.23 mg QUE/g DW (Table 1). The BA extract exhibited the highest TFC, followed by AP, CaA, winged bean (*Psophocarpus tetragonolobus* (L.) D.C.; PT), and MoC extracts, respectively. The HC extract had the lowest TFC (2.1 mg QUE/g DW). Moreover, this study found that the TFC was lower than the TPC overall.

#### 2.1.3. Phenolic Compounds Analyzed by HPLC-DAD

The phenolic compounds in 15 northeastern Thai edible plant extracts were quantitatively analyzed using HPLC-DAD. The total phenolic contents ranged from 20.79 to 3216.88 μg/g DW across the extracts, as shown in Table 2. The most varied phenolic profiles were found in HC, rice paddy herb (*Limnophila aromatica* (Lam.); LA), and MaC extracts, which consisted of 10 phenolic acids and flavonoids, except either ferulic acid (FA) or rutin (RN). Caffeic acid (CA) was the phenolic acid most commonly found among the extracts, followed by protocatechuic acid (PCCA), sinapic acid (SA), and RN. The phenolic with the highest content was chlorogenic acid (ChA), at 3109 μg/g DW, in Thai eggplant (*Solanum xanthocarpum* Schrad. & Wendl.; SX) extract. The gallic acid (GA) content ranged from 1.55 to 1921.40 μg/g DW, with the highest content being detected in the CaA extract. *p*-hydroxybenzoic acid (*p*-HO) was only detected in four extracts, ranging from 241.77 to 349.02 μg/g DW, mainly in the LA extract. The PCCA contents ranged between 6.81 and 452.38 μg/g DW, being particularly rich in the PT extract, followed by the HC, LA, PO, and SX extracts. The green amaranth (*Amaranthus viridis* L.; AV) extract showed the lowest PCCA content. Syringic acid (SyA) was found in 7 extracts, varying from 11.05 to 578.61 μg/g DW. The CI extract had the highest SyA content, followed by the HC, MaC, AP, and asiatic pennywort (*Centella asiatica* (L.) Urban; CeA) extracts as well as the AV and LA extracts. The PO extract presented the highest contents of both sinapic acid (SA) and quercetin (QU) at 507.96 and 60.35 μg/g DW, respectively. The yellow velvetleaf (*Limnocharis flava* (L.) Buchenau; LF) extract exhibited the highest ferulic acid (FA) content at 34.84 μg/g DW. The contents of *p*-coumaric acid (*p*-CA) and rutin (RN) were also found to be high in the BA extract, at 20.87 and 1659.90 μg/g DW, respectively. Among the 15 extracts, GA, *p*-HO, PCCA, SyA, ChA, CA, SA, *p*-CA, RN, and QU were detected in the HC and MaC extracts. Liu [20] and Khanam et al. [21] reported that hydroxybenzoic acids (GA, *p*-HO, PCCA, and SyA) and hydroxycinnamic acids (*p*-CA and FA) were the most abundant in plant foods and leafy vegetables. The current findings provide additional evidence that Thai local edible plants are a rich source of phytochemical compounds. These compounds in edible plants and mixtures or combinations of vegetables and fruits had potent biological properties, such as antioxidant and anticancer effects and the ability to prevent degenerative diseases such as type 2 diabetes [20,22,23]. Especially, the top 3 extracts contained high contents of phenolic compounds, with the SX extract exhibiting the highest contents of ChA and CA, whereas GA was reported to be the highest in the CaA extract. High contents of tryptophan, SA, and QU were found in the PO extract.

### 2.2. Biological Activities

#### 2.2.1. Antioxidant Activities

The antioxidant capacities of the 15 northeastern Thai local edible plant extracts were examined at a concentration of 50 μg/mL using ABTS, DPPH, and FRAP assays. The ABTS inhibition of the plant extracts ranged widely, from 9.63% to 100%, while Trolox, the positive control, showed ABTS inhibition of 89.71% (Table 3). The CaA, LA, and PO extracts exhibited significantly higher ABTS inhibition than Trolox. ABTS inhibition higher than 50% was found for the CaA, LA, HC, AP, PO, MaC, and CeA extracts. The CaA extract showed the best IC_50_ value, at 9.98 μg/mL, followed by the HC (34.40 μg/mL), AP (43.27 μg/mL), PO (55.10 μg/mL), MaC (59.98 μg/mL), and CeA (73.02 μg/mL) extracts, respectively. According to the classification of antioxidant power by Kusumawati et al. [24], the CaA, HC, and AP extracts showed very strong ABTS antioxidant activity (IC_50_ < 50 μg/mL), while the PO, MaC, and CeA extracts showed strong ABTS antioxidant activity (IC_50_ = 50–100 μg/mL). For the DPPH assay, the inhibition ranged from 7.21% to 95.18%, while the DPPH inhibition for Trolox was 95.90%. The CaA, LA, and PO extracts showed DPPH inhibition higher than 50%. The FRAP values for the extracts were 0.08 to 253.92 mM Fe^2+^/100 g DW, whereas that of Trolox was 4649.69 mmol Fe^2+^/100 g DW. The highest IC_50_ value was also found for the CaA extract, at 5.53 μg/mL. The IC_50_ of the LA extract was 13.16 μg/mL, and that of the PO extract was 65.63 μg/mL; moreover, the 3 extracts were classified as having very strong DPPH antioxidant activity. The FRAP values of the extracts were 0.08 to 253.92 mmole Fe^2+^/100 g DW, whereas that of Trolox was 4649.69 mmol Fe^2+^/100 g. High FRAP was found for the CaA extract (253.92 mmol Fe^2+^/100 g DW) and LA (109.92 mmol Fe^2+^/100 g DW), with capacities about 18- to 43-fold lower than that of Trolox. These findings demonstrated that the CaA extract showed the best antioxidant activity in the DPPH, ABTS, and FRAP assays, followed by the LA and PO extracts.

#### 2.2.2. α-Glucosidase-Inhibitory (AGI) Activity

The α-glucosidase-inhibitory (AGI) activity, reported as the IC_50_ value, varied from 0 to higher than 1 mg/mL across the extracts. Out of the 15 extracts, 10 exhibited IC_50_ values indicating AGI, as presented in Table 3. Among the edible vegetable extracts, the LF extract revealed an IC_50_ value higher than 1 mg/mL, whereas the best value was found for the CaA extract at 0.13 µg/mL. According to the IC_50_ values, the extracts ranked as follows in terms of AGI—PO, MaC, and HC—but with no statistical significance (*p* < 0.05). The MoC extract exhibited significantly stronger AGI activity (IC_50_ = 16 µg/mL) than the LA extract (IC_50_ = 198 µg/mL). Acarbose (IC_50_ = 211 µg/mL), a standard compound, showed similar AGI activity to the SX extract (IC_50_ = 212 µg/mL). The BA, BR, CI, and PT extracts could not inhibit α-glucosidase in this study. Interestingly, the extracts of four edible plants—wild guava (CaA), Vietnamese coriander (PO), water clover (MaC), and fish mist (HC)—demonstrated significantly promising α-glucosidase-inhibitory (AGI) activity, which was higher than that of the standard α-glucosidase inhibitor drug (acarbose). The highest AGI found for the CaA extract was 1600 times greater than that of acarbose. Our finding is supported by Kamble et al. [25], who found that a crude chloroform *Careya arborea* (CaA) extract had the potential to inhibit α-amylase. According to the phenolic profile (Table 2), the major phenolic acid and flavonoid compound in the CaA extract was gallic acid (GA), while the major compounds in the PO extract were sinapic acid (SA) and quercetin (QU). Moreover, the MaC and HC extracts contained *p*-HO as a main phenolic acid. Several reports stated that GA and QU exhibited much higher α-glucosidase-inhibitory activity than acarbose [26,27,28,29]. Similar to our results, CaA and PO extracts with abundant GA and QU also showed the highest AGI activity among all extracts.

### 2.3. Principal Component Analysis (PCA) and Clustering Analysis

**Figure 1:**
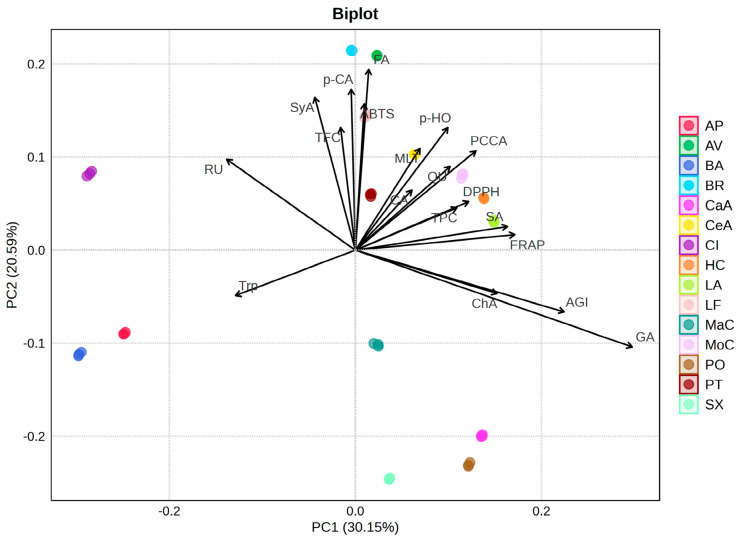
PCA biplot between the selected PCs of 15 northeastern Thai local edible vegetable extracts.

An unsupervised PCA, multivariable data analysis, was conducted for the relationship between the contents of phytochemical compounds, antioxidants, and α-glucosidase inhibitory (AGI) activities and the 15 Thai edible plant extracts. The PCA biplot consisted of two PCA components (PCs), with PC1 and PC2 accounting for 50.74% of the total variance (30.15% and 20.59%, respectively), as shown in Figure 1. The 15 Thai local edible plant extracts were distributed across four segregated areas based on their phenolic compounds, antioxidants, and AGI activities. The antioxidant activities (ABTS, DPPH, and FRAP assays) and the compounds SA, PCCA, *p*-HO, *p*-CA, FA, and QU were positioned on the positive sides of PC1 and PC2. The antioxidant activities showed a strong correlation with the TPC, TFC, GA, CA, SA, QU, and AGI. Our results are in accordance with the strong relationship between TPC and antioxidant activity in vegetables [30,31] and Chinese medicinal plants [32]. The greater distance on PC1 of GA and ChA helped to separate the CaA, PO, SX, and MaC extracts from the others, depending on the highest GA and ChA contents. The GA had a high–strong correlation with AGI and TPC and also moderately correlated with antioxidant activities. AGI showed strong relationships with antioxidant activities and the contents of GA and SA, while tryptophan and rutin were negligibly correlated with AGI. Therefore, the BA, CI, and AP extracts were separated and were accumulated negatively on PC1.

**Figure 2:**
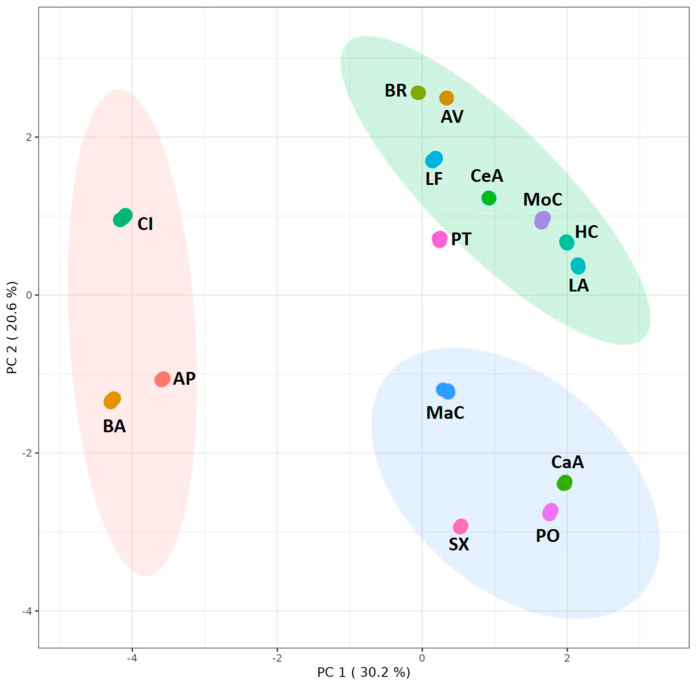
Clustering results obtained using K−means analysis.

The data set of phytochemical compounds and the antioxidant and AGI activities of the 15 Thai local edible plant extracts were analyzed using K-means clustering. The clustering not only enabled a better observation between the phytochemical compounds, antioxidants capacities, and AGI of the 15 extracts but also confirmed the results derived from the PCA. Based on the correlation of phenolic compounds and their activities, the analysis was grouped into 3 clusters as presented in Figure 2. The CaA, PO, SX, and MaC extracts were classified as Cluster I, which showed high AGI and antioxidant activities as well as high contents of MLT, TPC, GA, QU, ChA, and SA. Cluster II consisted of the CeA, LA, MoC, HC, AV, BR, PT, and LF extracts, showing a slight decrease in AGI and antioxidant activities compared to Cluster I, and high levels of SA, PCCA, *p*-HO, *p*-CA, FA, and QU. In Cluster III, the BA, AP, and CI extracts showed the highest levels of rutin and tryptophan, but no AGI activity.

### 2.4. Network Pharmacology

#### 2.4.1. Compounds and Therapeutic Targets Related to T2DM

**Figure 3:**
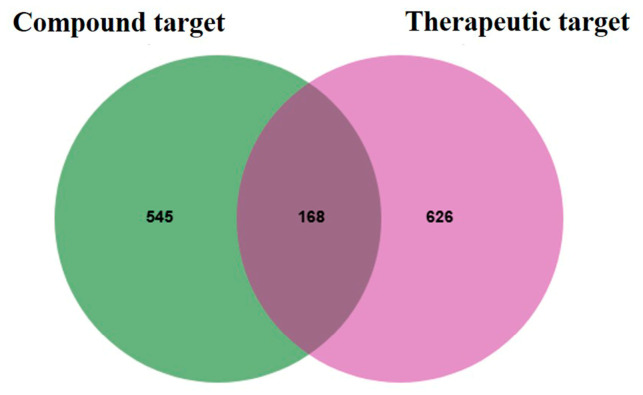
Venn diagram showing compounds and therapeutic targets associated with T2DM.

The compounds and therapeutic targets were predicted prior to analyzing the antidiabetic mechanism. The STP and SEA databases showed 714 potential compound targets, and 794 T2DM-related gene targets were found in the GeneCards database. Duplicated compound-targets were excluded. The Venn diagram analysis showed the presence of 168 overlapping targets directly related to polyphenols and T2DM (Figure 3).

#### 2.4.2. Protein–Protein Interaction (PPI) Network

**Figure 4:**
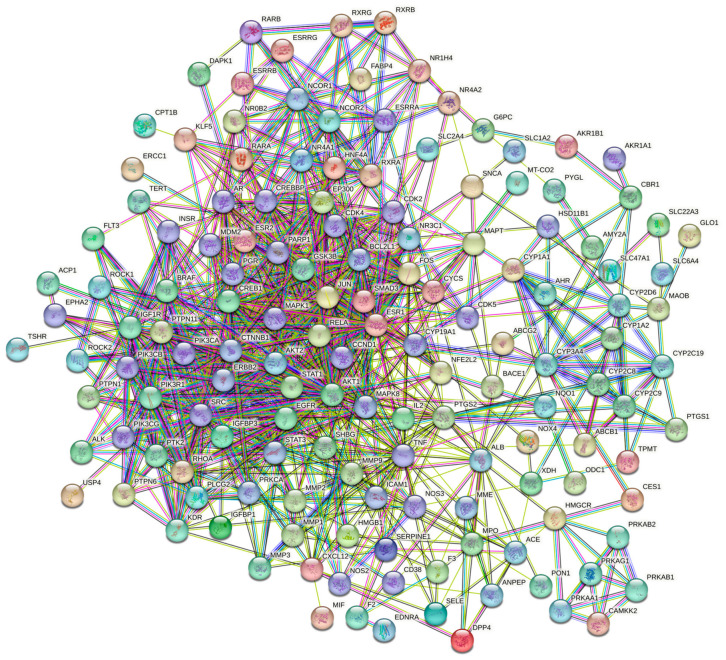
Original protein–protein interaction (PPI) network constructed using the STRING database, showing the overlapping targets between the polyphenols in the Thai local edible plant extracts and T2DM.

A network of phytochemical compounds and their T2DM-related targets was constructed to investigate the potential antidiabetic mechanism. Then, the 168 polyphenol–T2DM overlapping targets were imported into the online STRING database to construct the PPI network. The STRING results revealed 166 nodes connected to a network with 906 edges, and the 19 disconnected nodes were not shown in this network. The average node degree was 10.9, while the average local clustering coefficient and the PPI enrichment *p*-value were 0.454 and <1.0 × 10^−16^, respectively. According to the results, the edge of this network has significantly more interactions (n = 906) than expected (n = 253), implying that those protein targets have more functional and physical interactions among themselves (Figure 4).

The identified hub targets involved in polyphenol-related antidiabetic treatment were predicted by using three methods—the degree, betweenness, and closeness methods—to ensure accuracy. The overlapping hub targets consisted of RAC-alpha serine/threonine-protein kinase (AKT1), catenin beta-1 (CTNNB1), epidermal growth factor receptor (EGFR), signal transducer and activator of transcription 3 (STAT3), estrogen receptor (ESR1), and tumor necrosis factor (TNF), as listed in Table 4.

#### 2.4.3. Gene Ontology (GO), Kyoto Encyclopedia of Genes and Genomes (KEGG) Enrichment Pathway, and Pathway–Compound–Target Interaction Analysis

**Figure 5:**
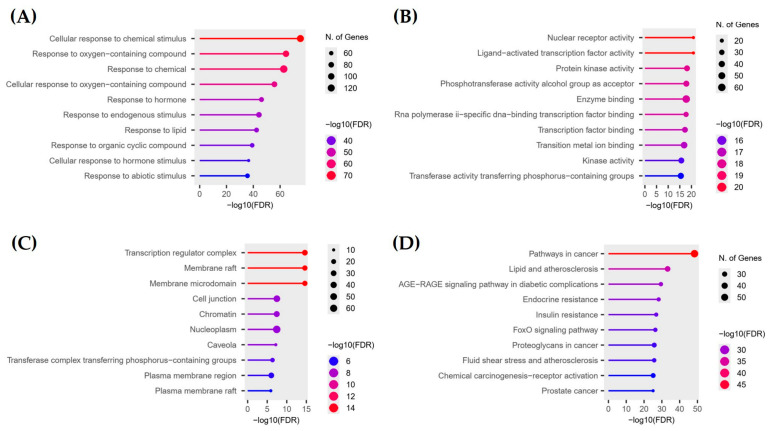
Top 10 enrichment analysis of the overlapping targets between the polyphenols in 15 northeastern Thai local edible plant extracts and T2DM. GO enrichment includes (**A**) biological process, (**B**) molecular function, (**C**) cellular component, and (**D**) KEGG pathway. The ShinyGO database was used for visualization.

The overlapping targets between the polyphenols in the Thai local edible plant extracts and antidiabetic effects were used to perform GO and KEGG enrichment analysis through the ShinyGO database [33]. The GO enrichment divided them into three categories, revealing 1447 biological processes (BP), 155 molecular functions (MF), and 85 cellular components (CC). The top 10 BP revealed cellular response to chemical stimulus, oxygen-containing compound, and hormone stimulus as well as response to chemical, hormone, lipid, and abiotic stimulus (Figure 5A). The top 10 MF revealed the activities of nuclear receptor, protein kinase, kinase, and a phosphotransferase activity alcohol group as acceptor (Figure 5B). Meanwhile, the top 10 CC exhibited membrane raft and microdomain, cell junction, chromatin, nucleoplasm, and plasma membrane region (Figure 5C). In addition, 189 pathways were obtained in KEGG pathway analysis, and the top 10 KEGG pathways were selected based on the false discovery rate (FDR) enrichment value. The most significant enriched KEGG pathways consisted of pathway in cancer, lipid and atherosclerosis, AGE-RAGE signaling pathway in diabetic complications, endocrine resistance, and insulin resistance, respectively (Figure 5D). Based on the potential AGI activity, the observed interaction of the polyphenols in Thai local edible plant genes and the T2DM genes indicated a potential impact of polyphenols in T2DM. The most relevant pathways involved in the extracts for T2DM treatment were found to be the insulin resistance and AGE-RAGE signaling pathways.

**Figure 6:**
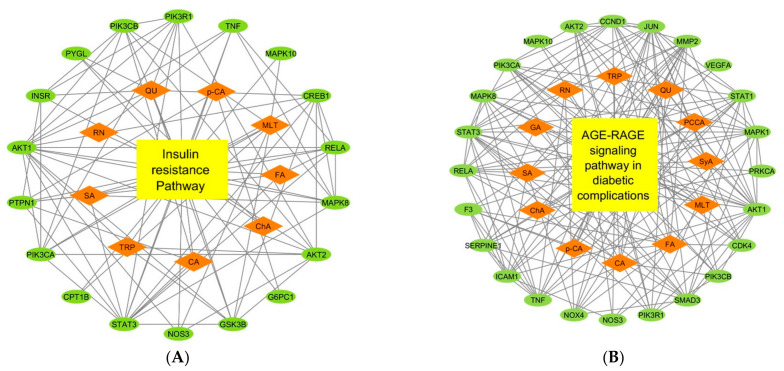
Gene–gene interactions in (**A**) insulin resistance pathway (hsa04931) and (**B**) AGE-RAGE signaling pathway in diabetic complications (hsa04933). Pathway–compound–target interaction of polyphenols of northeastern Thai local edible plant extracts (orange) with insulin resistance pathway (yellow) and target genes (green).

**Figure 7:**
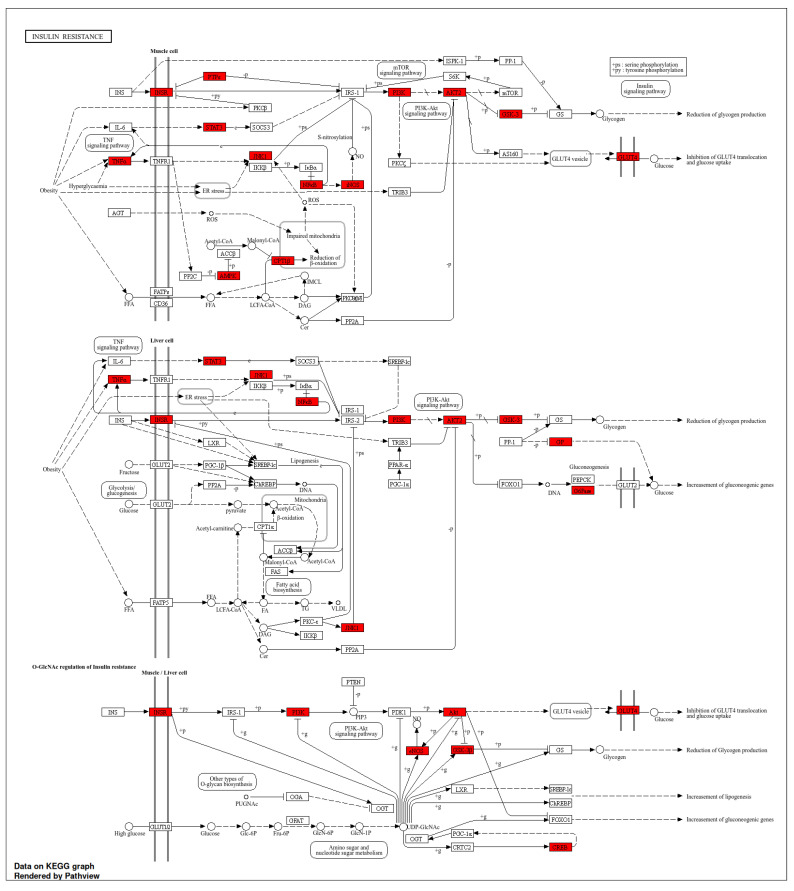
Insulin resistance pathway (hsa04931). Pathway map of polyphenols in 15 northeastern Thai local edible plants in the treatment of type 2 diabetes mellitus. The main targets that are involved in the diabetes-related pathway are highlighted in red. Arrows represent activation effects, and T-arrows represent inhibitory effects. The ShinyGO database was used for visualization.

According to network pharmacology, the related targets of the insulin resistance pathway included CPT1B, CREB1, AKT1, AKT2, G6PC1, GSK3B, INSR, NOS3, PIK3CA, PIK3CB, PIK3R1, MAPK8, MAPK10, PTPN1, PYGL, RELA, STAT3, and TNF, as listed in Table 5. Among all the compounds, only 9 were involved in the insulin resistance pathway (Figure 6A). Melatonin (MLT) and quercetin (QU) had the highest interaction with those insulin resistance-related genes, followed by sinapic acid (SA), tryptophan (TRP), caffeic acid (CA), ferulic acid (FA), *p*-coumaric acid (*p*-CA), chlorogenic acid (ChA), and rutin (RN), respectively. Figure 7 displays the insulin resistance pathway related to the multiple mechanisms. Polyphenols from the 15 Thai edible plants were able to decrease the activation of signaling molecules including PI3K and AKT via the mTOR signaling pathway; the PI3K gene was modulated by melatonin, quercetin, and caffeic acid, and the AKT gene by quercetin. Furthermore, rutin regulated oxidative stress, the accumulation of intracellular lipid derivatives, and inflammation by modulating TNF. Melatonin was predicted to be a target of GLUT4, while tryptophan was predicted to be a target of glycogen synthase kinase 3 (GSK3). Insulin resistance was affected by a reduction in glucose transporter 4 (GLUT4) translocation, causing glucose and glycogen synthesis in skeletal muscle, increased hepatic gluconeogenesis, and reduced glycogen synthesis in the liver [34,35,36].

**Figure 8:**
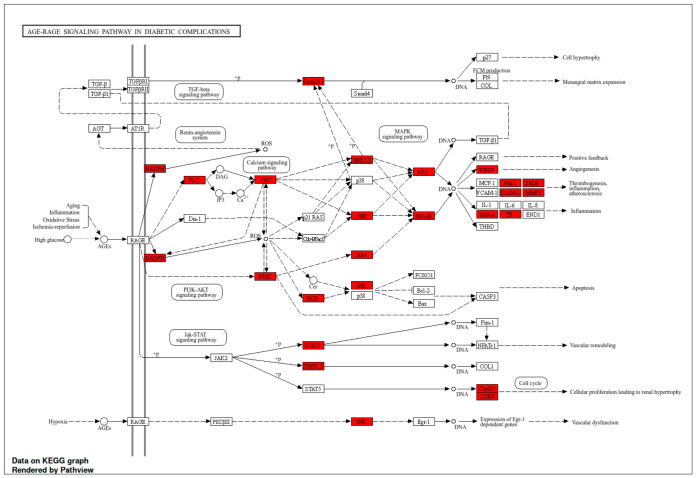
AGE-RAGE signaling pathway in diabetic complications (hsa04933). Pathway map of polyphenols in northeastern Thai local edible plants in the treatment of type 2 diabetes mellitus. The main targets that are involved in the diabetes-related pathway are highlighted in red. Arrows represent activation effects, and T-arrows represent inhibitory effects. The ShinyGO database was used for visualization.

A complication of diabetes is the activation of the AGE-RAGE signaling pathway by a complex group of compounds produced via the non-enzymatic glycation and oxidation of proteins due to hyperglycemia [37,38]. In this study, the network pharmacology showed that the targets related to this pathway included CDK4, AKT1, AKT2, F3, ICAM1, JUN, SMAD3, MMP2, NOS3, NOX4, SERPINE1, PIK3CA, PIK3CB, PIK3R1, PRKCA, MAPK1, MAPK8, MAPK10, CCND1, RELA, SELE, STAT1, STAT3, TNF, and VEGFA, as listed in Table 5. The PPI analysis in this study found that 12 polyphenols interacted with the target genes associated with the AGE-RAGE signaling pathway in diabetic complications (Figure 6B). Interestingly, sinapic acid had the most interactions with the targets, which consisted of NOX4, MAPK8, RELA, STAT3, F3, ICAM1, JUN, and MMP2, followed by ferulic acid, melatonin, and caffeic acid, as well as quercetin. Tryptophan and *p*-coumaric acid showed greater interactions in this network, and also interacted with gene targets related to the insulin resistance pathway. In addition, chlorogenic acid, rutin, syringic acid, gallic acid, and protocatechuic acid had several interactions with gene targets. The AGE-RAGE signaling also activated several intracellular signaling pathways such as NADPH oxidase, protein kinase C, and mitogen-activated protein kinases (MAPKs) that are associated with inflammation via nuclear factor-kappa B (NF-κB) activity. In addition, NF-κB could be directly activated by the excess of multiple inflammatory factors and cytokines such as TNF-α and VEGF (Figure 8). The Janus kinase (JAK)/signal transducers and activators of the transcription (STAT) signaling pathway are associated with inflammation and have been found to promote NF-κB cascade. Therefore, our network pharmacology results may suggest that polyphenols in Thai local edible plants have the potential to not only inhibit α-glucosidase activity but also treat T2DM and its related complications.

## 3. Materials and Methods

### 3.1. Chemicals and Reagents

Methanol and acetonitrile were products of ACI Labscan (Bangkok, Thailand). Standard chemicals, HPLC-grade, including tryptophan, serotonin, gallic acid (GA), protocatechuic acid (PCCA), *p*-hydroxybenzoic acid (*p*-HO), syringic acid (SyA), caffeic acid (CA), chlorogenic acid (ChA), *p*-coumaric acid (*p*-CA), ferulic acid (FA), sinapic acid (SA), rutin (RN), and quercetin (QU), were purchased from Sigma-Aldrich (St. Louis, MO, USA). Standard melatonin was obtained from Shanghai Chemical (Shanghai, China). Folin–Ciocalteu reagent was obtained from Merck (Darmstadt, Germany). 2,2′-Azinobis (3-ethylbenzothiazoline-6-sulfonic acid) disodium salt (ABTS), 2,2-diphenyl-1 picrylhydrazyl radical (DPPH), 2,4,6-Tris(2-pyridyl)-1,3,5-triazine (TPTZ), aluminum chloride hexahydrate, and 6-hydroxy-2,5,7,8-tetramethyl chromane 2-carboxylic acid (Trolox) were purchased from Sigma Aldrich (St. Louis, MO, USA). α-Glucosidase enzyme (EC 3.2.1.20, Cas No. 9001-42-7) was purchased from Megazyme (Wicklow, Ireland), *p*-nitrophenyl α-D-glucopyranoside (*p*NPG) was obtained from Tokyo Chemical Industry Co., Ltd. (Tokyo, Japan), and acarbose was obtained from FUJIFILM Wako Pure Chemical Corporation (Osaka, Japan).

### 3.2. Sample Extraction

Fifteen northeastern Thai local edible plants were purchased from the market in Mahasarakham province, Thailand, in 2023 as listed in Table 6. The edible plants were identified by Dr. Wanida Caichompoo, Faculty of Pharmacy, Mahasarakham University, Thailand. Voucher specimens were deposited at the Faculty of Pharmacy, Mahasarakham University, Thailand. The edible plants were dried using a Lyolab^®^ freeze-drying chamber (Lyophilization Systems Inc., Kingston, NY, USA) and ground into fine powder using a grinder prior to extraction. Dried edible plant powder (20 g) was extracted with absolute methanol in a proportion of 1:10 (*w*/*v*) by ultrasonication (Transsonic T460/H, Elma GmbH, Singen, Germany) for 30 min [39]. The sample was then filtered using Whatman No.1 paper, and the residue was double-extracted. The filtrate was evaporated on a rotary evaporator (Rotavapor^®^ R-100, Buchi, Switzerland) at 40 °C and stored at −20 °C.

### 3.3. Phytochemical Contents

#### 3.3.1. Tryptophan, Serotonin, and Melatonin Contents Analyzed by High-Performance Liquid Chromatography (HPLC)

To quantify the contents of tryptophan, serotonin, and melatonin in each sample, it was necessary to purify the samples by solid-phase extraction, using a Strata^®^ C_18_-E cartridge, Phenomenex (Torrance, CA, USA). The tryptophan, serotonin, and melatonin were identified by High-Performance Liquid Chromatography (Agilent 1260, Santa Clara, CA, USA) equipped with a fluorescence detector (FD) following our previous study [40]. Each experiment was performed in triplicate.

#### 3.3.2. Determination of Total Phenolic and Flavonoid Contents

The total phenolic contents (TPCs) were determined using Folin–Ciocalteu reagent according to Um et al. [41] with some modifications. The extracts (20 μL) were mixed with 7% sodium carbonate (80 μL) and 10% Folin–Ciocalteu reagent (100 μL). The mixture was incubated in darkness for 30 min and then measured at a wavelength of 760 nm using a Sunrise^TM^ microplate reader (Tecan Trading AG, Männedorf, Switzerland). The results are expressed as mg of gallic acid equivalent (GAE) per gram dry weight, for triplicate assays. The total flavonoid contents (TFCs) were estimated using the aluminum chloride (AlCl_3_) colorimetric method [42]. Briefly, a mixture of 100 μL of extract and 100 μL of 2% AlCl_3_ solution was incubated in darkness for 30 min. Then, the absorbance of the mixture was measured using a microplate reader at 415 nm. The TFCs are reported as mg of quercetin equivalent (QUE) per gram dry weight, for triplicate assays.

#### 3.3.3. Phenolic Compound Analysis by HPLC-DAD

The extracts were filtered through 0.45 µm PTFE syringe filters and analyzed by HPLC (Shimadzu Corp., Kyoto, Japan) with a UV diode array detector (DAD). The phenolics profile was analyzed according to our previous study [40]. The column used was a Unisol^®^ C_18_ column (4.6 mm × 250 mm, 5 µm). A gradient system at a flow rate of 0.8 mL/min was used with 1% acetic acid as mobile phase A and acetonitrile as mobile phase B. Peaks were monitored at 280, 320, and 380 nm, respectively. The retention times of the extracts were identified by comparing the retention times with standard phenolic acids including gallic acid (GA), protocatechuic acid (PCCA), syringic acid (SyA), *p*-hydroxybenzoic acid (*p*-HO), and caffeic acid (CA) at 280 nm; chlorogenic acid (ChA), *p*-coumaric acid (*p*-CA), ferulic acid (FA), and sinapic acid (SA) at 320 nm; and rutin (RN) and quercetin (QU) at 370 nm. The standard phenolics and extracts were assayed in triplicate.

### 3.4. Antioxidant Activities

The antioxidant capacities of the 15 Thai local edible plant extracts were assayed using the ABTS, DPPH, and FRAP methods. Each test sample was dissolved in methanol to make a final concentration of 50 µg/mL, and then samples showing ABTS and DPPH inhibition greater than 50% were selected to determine the IC_50_ values. Trolox was used as a reference standard for comparison in all assays, and each assay was performed in three replicates.

For the ABTS assay, the ABTS reagent consisted of a mixture of 7 mM ABTS and 140 mM potassium persulfate, and involved incubation for 12–16 h in darkness. The reagent was diluted with distilled water in a 1:15 *v*/*v* ratio. The extract (100 μL) was mixed with 100 μL of ABTS reagent and left for 30 min. The absorbance at 415 nm was measured using a Sunrise^TM^ microplate reader (Tecan Trading AG, Männedorf, Switzerland) [43]. The percentage inhibition was calculated by using Equation (1):

(1) $$Inhibition\left(\%\right)=\frac{\left(Absorbance\;of\;Control-Absorbance\;of\;Sample\right)}{Absorbance\;of\;Control}\times 100$$

The extract concentration resulting in 50% inhibition (the IC_50_ value) was calculated from a plot of percentage ABTS inhibition (%) and extract concentration (μg/mL).

In the DPPH assay, the DPPH radical scavenging ability was determined using the method of Arabshahi-Delouee and Urooj [44]. A mixture of the extract and 200 µM DPPH reagent in a proportion of 100 μL:100 μL was left to stand for 30 min in darkness. The absorbance was measured at 517 nm using a microplate reader. The DPPH capacity is presented as percentage inhibition for all extracts, and IC_50_ values for only the extracts that showed inhibition higher than 50%.

The ferric reducing antioxidant power (FRAP) assay was performed according to a procedure published by Iqbal et al. [45]. The FRAP reagent included the following: 300 mM acetate buffer (pH 3.6), ferric chloride solution (20 mM), and 10 mM 2,4,6-Tris (2-pyridyl)-1,3,5-triazine (TPTZ) solution in a proportion of 10:1:1 (*v*/*v*/*v*). The FRAP reagent was warmed at 37 °C for 30 min before use. In a 96-well plate, the extract (20 μL) was mixed with 80 μL of FRAP reagent and left to stand for 5 min, and the absorbance was measured at 595 nm. Ferrous sulfate was used to generate the standard curve. The FRAP value is expressed as mmol Fe^2+^/100 g DW.

### 3.5. α-Glucosidase-Inhibitory (AGI) Activity

The α-glucosidase-inhibitory (AGI) activity was assayed for all extracts according to Kicel et al. [46] with slight modifications. Briefly, the test samples were prepared at a concentration of 1 mg/mL. The test samples (50 µL) were mixed with 15 µL of α-glucosidase solution (0.5 U/mL) and 20 µL of 100 mM phosphate buffer (pH 6.8) in a 96-well plate. The plate was pre-incubated at 37 °C for 10 min. Then, 15 µL of 5 mM p-nitrophenyl-α-D-glucopyranoside (pNPG) substrate was added to each well and incubated at 37 °C for 15 min. The enzymatic reaction product, p-nitrophenol (yellow), was measured according to the absorbance at 405 nm, determined using a Sunrise^TM^ microplate reader (Tecan Trading AG, Männedorf, Switzerland). Acarbose and 100 mM phosphate buffer (pH 6.8) were used as a positive and negative control, respectively. The experiments were conducted in triplicate, and the percentage α-glucosidase-inhibitory activity was calculated using Equation (2):

(2) $$AGI\;activity(\%)=\frac{\left(Absorbance\;of\;Control-Absorbance\;of\;Sample\right)}{Absorbance\;of\;Control}\times 100$$

The AGI activity is expressed as the IC_50_ value, which is the concentration of the extract required to inhibit 50% of the enzyme’s activity in the assay.

### 3.6. Multivariate Data Analysis

Multivariate data analysis was performed using MetaboAnalyst 6.0, a web-based statistical platform (https://www.metaboanalyst.ca, accessed on 21 April 2025). Unsupervised principal component analysis (PCA) was performed using the prcomp package to reduce dimensionality and minimize the data. The data, including the TPC, TFC, indolamines (melatonin and tryptophan), and phenolic compounds analyzed via HPLC, and the activities of antioxidants and α-glucosidase inhibition, were subjected to data normalization methods, including data normalization by sum, Log10 transformation, and data scaling by Pareto scaling. The clustering was performed with the K-means function in the package stat.

### 3.7. Network Pharmacology Prediction

#### 3.7.1. Prediction of Compound and Therapeutic Targets of Type 2 Diabetes Mellitus (T2DM)

The screening of therapeutic targets in T2DM was performed in the same manner as our previous study [47]. Briefly, the targets of the phenolic compounds analyzed by HPLC—i.e., GA, PCCA, *p*-HO, SyA, CA, ChA, *p*-CA, FA, SA, RN, QU, MLT, and TRP—were searched for using SwissTargetPrediction (http://www.swisstargetprediction.ch/, accessed on 18 November 2025) and Similarity Ensemble Approach (https://sea.bkslab.org/, accessed on 18 November 2025). For therapeutic targets, the T2DM-related targets were obtained from the GeneCards database (https://www.genecards.org/, accessed on 25 November 2025). The search term was previously set using “antidiabetic” as a keyword. The UniProt IDs corresponding to *Homo sapiens* were selected. Subsequently, the compound–therapeutic targets were mapped by using the online Jvenn tool (https://jvenn.toulouse.inrae.fr/app/example.html, accessed on 25 November 2025). All the collected targets were combined, and any potential duplication was removed before continuing to the next step. The overlapping compounds and therapeutic targets were selected for further protein–protein interaction network analysis.

#### 3.7.2. Protein–Protein Interaction (PPI) Network

The Search Tool for the Retrieval of Interacting Genes/Proteins (SRING) database (https://string-db.org/, accessed on 25 November 2025) was employed to predict the protein–protein interaction (PPI) network of the overlapping T2DM targets related to polyphenols in Thai local edible plants. The PPI parameter set was as follows: *H. sapiens* was selected as an organism, evidence for network edges was restricted to high-confidence interactions (score > 0.7), and disconnected nodes were excluded from the network [48]. The STRING data were exported to Cytoscape (version 3.10.4) to visualize the PPI network. Subsequently, the CytoHubba-plugin in Cytoscape software was used to screen the hub targets based on degree, betweenness, and closeness methods.

#### 3.7.3. Gene Ontology (GO), Kyoto Encyclopedia of Genes and Genomes (KEGG) Enrichment Pathway, and Pathway–Compound–Target Interaction Analysis

The GO enrichment and KEGG pathway analysis identified key T2DM-related signaling pathways associated with the overlapping compound and therapeutic targets, using the ShinyGO database version 0.85.1 (https://bioinformatics.sdstate.edu/go/, accessed on 2 December 2025). The top 10 GO enrichment and KEGG pathways were set with a false discovery rate (FDR) cutoff of 0.05 [33]. The ShinyGO database was used to visualize all pathways. The T2DM-related pathway was further selected for pathway–compound–target interaction analysis. Pathway–compound–target interactions were constructed using Cytoscape software to explore the correlation and visualization of the interactions.

### 3.8. Statistical Analysis

All data are reported as mean ± SD. Statistical analysis was performed using the IBM SPSS Statistics v.29 program. The data were compared using one-way analysis of variance (ANOVA) followed by Tukey’s HSD test; a *p*-value less than 0.05 was considered to indicate a statistically significant difference.

Data visualization was performed as follows: A Venn diagram was prepared using the online Jvenn tool (https://jvenn.toulouse.inrae.fr/app/example.html, accessed on 25 November 2025), the PCA was performed through the MetaboAnalyst 6.0 platform (https://www.metaboanalyst.ca, accessed on 21 April 2025), the GO and KEGG enrichment analysis and pathway mapping were performed using the ShinyGO database version 0.85.1. (https://bioinformatics.sdstate.edu/go/, accessed on 2 December 2025), and the pathway–compound–target interactions were constructed using Cytoscape software.

## 4. Conclusions

Among the sample tests, four edible plants—wild guava (CaA), Vietnamese coriander (PO), water clover (MaC), and fish mist (HC)—demonstrated significant α-glucosidase-inhibitory (AGI) activity, surpassing the standard drug, acarbose. Notably, the CaA extract exhibited the highest AGI activity, being 1000 times more potent than acarbose. Our results show that the phenolic levels correlated closely with AGI activity, with gallic acid, sinapic acid, and quercetin reaching their highest concentrations in the CaA, rice paddy herb (LA), and PO extracts, respectively. Based on network pharmacology predictions, these edible plants show potential in managing T2DM by modulating insulin resistance and AGE-RAGE signaling pathways. These mechanisms are primarily attributed to the phenolic contents of the plants, suggesting that these local edible Thai plants serve as viable natural alternatives for the prevention and management of T2DM.

## Figures and Tables

**Table 1:** The contents of tryptophan and melatonin, and total phenolic content (TPC) and total flavonoid content (TFC) of 15 northeastern Thai local edible plant extracts.

Plant Extract Code	Tryptophan (ng/g DW)	Melatonin (ng/g DW)	TPC (mg GAE/g DW)	TFC (mg QUE/g DW)
AP	2138.78 ± 2.78 ^b^	ND	174.38 ± 7.28 ^c^	14.83 ± 0.05 ^b^
AV	ND	ND	12.69 ± 0.51 ^k^	4.10 ± 0.05 ^hi^
BA	2213.05 ± 14.04 ^a^	ND	51.15 ± 0.64 ^hi^	16.23 ± 0.10 ^a^
BR	ND	ND	9.36 ± 0.31 ^l^	3.47 ± 0.08 ^j^
CaA	ND	ND	606.77 ± 6.99 ^a^	12.21 ± 0.18 ^c^
CeA	ND	ND	93.76 ± 3.94 ^f^	2.36 ± 0.03 ^k^
CI	15.75 ± 0.55 ^e^	ND	23.27 ± 1.42 ^k^	4.28 ± 0.15 ^h^
HC	ND	4.38 ± 0.40 ^b^	121.07 ± 1.63 ^e^	2.11 ± 0.07 ^k^
LF	ND	ND	71.71 ± 1.44 ^g^	6.00 ± 0.05 ^f^
LA	ND	ND	246.00 ± 2.31 ^b^	3.74 ± 0.06 ^ij^
MaC	1693.40 ± 0.77 ^c^	99.17 ± 4.58 ^a^	37.69 ± 0.99 ^j^	2.28 ± 0.06 ^k^
MoC	23.14 ± 0.45 ^e^	2.44 ± 0.12 ^b^	41.55 ± 0.49 ^ij^	7.21 ± 0.16 ^e^
PO	985.56 ± 2.89 ^d^	ND	118.51 ± 4.10 ^e^	2.50 ± 0.19 ^k^
PT	ND	ND	55.52 ± 1.13 ^h^	11.04 ± 0.13 ^d^
SX	ND	ND	147.48 ± 3.92 ^d^	5.16 ± 0.18 ^g^

^a–l^ means within a column with the same superscript letter are not significantly different (*p* ≥ 0.05, n = 3) according to Tukey’s HSD multiple range comparison tests. ND means not detected. All values are shown in units of per gram dry weight (DW).

**Table 2:** Phenolic profiles found in 15 northeastern Thai local edible plant extracts by HPLC-DAD.

Plant Extracts	Phenolic Compounds (μg/g DW)											Total
	GA	*p*-HO	PCCA	SyA	ChA	CA	SA	*p*-CA	FA	RN	QU	(μg/g DW)
AP	ND	ND	ND	32.66 ± 0.61 ^c^	ND	26.22 ± 0.44 ^cd^	ND	ND	ND	551.75 ± 23.72 ^b^	47.45 ± 0.16 ^b^	658.08 ± 24.93
AV	ND	ND	6.81 ± 0.18 ^g^	12.85 ± 0.16 ^d^	ND	15.02 ± 0.08 ^fg^	7.83 ± 0.02 ^e^	ND	ND	30.33 ± 0.10 ^d^	11.69 ± 0.04 ^e^	84.53 ± 0.58
BA	ND	ND	14.27 ± 0.82 ^f^	ND	5.04 ± 0.07 ^g^	27.13 ± 1.48 ^c^	14.19 ± 0.19 ^e^	20.87 ± 1.33 ^a^	ND	1659.90 ± 138.90 ^a^	ND	1741.4 ± 142.79
BR	ND	ND	ND	ND	2.68 ± 0.00 ^g^	5.80 ± 0.09 ^h^	ND	ND	12.31 ± 0.24 ^d^	ND	ND	20.79 ± 0.33
CaA	1921.40 ± 10.21 ^a^	ND	ND	ND	ND	29.81 ± 0.38 ^b^	36.39 ± 2.10 ^e^	ND	ND	59.70 ± 0.57 ^d^	32.91 ± 0.11 ^c^	2080.21 ± 13.37
CeA	ND	256.41 ± 3.02 ^c^	27.16 ± 0.23 ^e^	15.29 ± 0.10 ^d^	197.80 ± 2.12 ^e^	16.90 ± 0.06 ^f^	182.54 ± 1.45 ^c^	17.47 ± 0.09 ^b^	ND	ND	44.76 ± 0.25 ^b^	758.33 ± 7.32
CI	ND	ND	ND	578.61 ± 10.56 ^a^	ND	ND	7.94 ± 0.02 ^e^	12.10 ± 0.11 ^c^	18.73 ± 0.21 ^b^	465.12 ± 9.02 ^b^	ND	1082.5 ± 19.92
HC	16.15 ± 0.25 ^de^	278.31 ± 2.46 ^b^	75.93 ± 1.53 ^b^	108.05 ± 3.02 ^b^	214.89 ± 3.91 ^d^	24.45 ± 0.48 ^de^	116.69 ± 1.23 ^d^	11.93 ± 0.26 ^c^	ND	293.89 ± 3.02 ^c^	27.78 ± 0.15 ^d^	1168.07 ± 16.31
LF	7.52 ± 0.19 ^ef^	ND	29.30 ± 1.30 ^de^	ND	ND	26.28 ± 0.45 ^cd^	ND	17.39 ± 0.22 ^b^	34.84 ± 0.54 ^a^	254.02 ± 5.90 ^c^	ND	369.35 ± 8.6
LA	3.91 ± 0.04 ^f^	349.02 ± 0.79 ^a^	71.44 ± 0.18 ^b^	11.05 ± 0.02 ^d^	249.41 ± 0.51 ^c^	12.95 ± 0.04 ^g^	249.21 ± 0.78 ^b^	18.18 ± 0.03 ^b^	13.97 ± 0.11 ^c^	ND	23.14 ± 0.04 ^d^	1002.28 ± 2.54
MaC	1.55 ± 0.02 ^f^	241.77 ± 1.93 ^d^	17.17 ± 0.29 ^f^	33.70 ± 0.28 ^c^	172.35 ± 1.32 ^f^	14.46 ± 0.17 ^g^	125.73 ± 0.88 ^cd^	5.47 ± 0.16 ^d^	ND	8.43 ± 1.94 ^d^	15.05 ± 0.05 ^e^	635.68 ± 7.04
MoC	15.79 ± 0.01 ^de^	ND	19.88 ± 0.25 ^f^	ND	ND	ND	57.22 ± 7.13 ^e^	ND	ND	31.72 ± 0.53 ^d^	ND	124.61 ± 7.92
PO	322.53 ± 2.63 ^b^	ND	61.06 ± 1.70 ^c^	ND	491.68 ± 16.04 ^b^	23.91 ± 3.07 ^e^	507.96 ± 62.18 ^a^	ND	ND	14.97 ± 1.13 ^d^	60.35 ± 5.34 ^a^	1482.46 ± 92.09
PT	39.46 ± 1.66 ^c^	ND	452.38 ± 5.86 ^a^	ND	ND	ND	ND	ND	14.35 ± 0.01 ^c^	21.78 ± 0.40 ^d^	28.20 ± 0.06 ^cd^	516.71 ± 7.99
SX	19.01 ± 0.10 ^d^	ND	34.42 ± 0.45 ^d^	ND	3109.46 ± 5.16 ^a^	33.85 ± 0.24 ^a^	20.14 ± 0.13 ^e^	ND	ND	ND	ND	3216.88 ± 6.08

^a–h^ means within a column with the same superscript letter are not significantly different (*p* ≥ 0.05, n = 3) according to Tukey’s HSD multiple range comparison tests. GA: gallic acid; *p*-HO: *p*-hydroxybenzoic acid; PCCA: protocatechuic acid; SyA: syringic acid; ChA: chlorogenic acid; CA: caffeic acid; SA: sinapic acid; *p*-CA: *p*-coumaric acid; FA: ferulic acid; RN: rutin; and QU: quercetin. ND means not detected.

**Table 3:** Antioxidant capacities and α-glucosidase-inhibitory activity of 15 northeastern Thai local edible plant extracts.

Plant Extract	ABTS Assay		DPPH Assay		FRAP Value	α-Glucosidase-Inhibitory Activity (IC_50_; µg/mL)
	Inhibition (%)	IC_50_ Value (μg/mL)	Inhibition (%)	IC_50_ Value (μg/mL)	(mM Fe^2+^/100 g DW)	
AP	61.34 ± 0.22 ^g^	43.27 ± 0.18 ^d^	19.75 ± 0.11 ^g^	-	52.69 ± 2.06 ^cd^	461.18 ± 4.49 ^g^
AV	6.66 ± 0.68 ^n^	-	11.40 ± 0.11 ^ij^	-	8.25 ± 0.21 ^d^	324.18 ± 4.39 ^e^
BA	23.04 ± 0.28 ^j^	-	11.87 ± 0.68 ^i^	-	13.83 ± 0.47 ^d^	ND
BR	9.63 ± 0.07 ^m^	-	10.14 ± 0.29 ^k^	-	4.42 ± 0.08 ^d^	ND
CaA	101.06 ± 0.11 ^a^	9.98 ± 0.02 ^b^	95.18 ± 0.00 ^a^	5.53 ± 0.00 ^b^	253.92 ± 4.47 ^b^	0.13 ± 0.00 ^a^
CeA	75.37 ± 1.13 ^e^	73.02 ± 0.68 ^g^	21.52 ± 0.44 ^f^	-	50.01 ± 0.68 ^cd^	376.09 ± 2.68 ^f^
CI	13.65 ± 0.94 ^l^	-	10.85 ± 0.48 ^ij^	-	0.08 ± 0.00 ^d^	ND
HC	69.02 ± 0.12 ^f^	34.40 ± 0.40 ^c^	39.73 ± 0.19 ^d^	-	43.40 ± 0.49 ^cd^	6.08 ± 0.2 ^a^
LF	15.45 ± 0.19 ^j^	-	11.56 ± 0.55 ^ij^	-	30.39 ± 0.29 ^d^	>1000 ^h^
LA	98.61 ± 0.07 ^b^	11.33 ± 0.05 ^b^	81.48 ± 0.29 ^b^	13.16 ± 0.67 ^c^	109.92 ± 6.41 ^c^	198.67 ± 0.51 ^c^
MaC	70.21 ± 0.73 ^f^	59.98 ± 0.60 ^f^	31.41 ± 0.11 ^e^	-	24.74 ± 0.57 ^d^	4.49 ± 0.03 ^a^
MoC	33.32 ± 0.27 ^j^	-	8.58 ± 0.71 ^kl^	-	12.90 ± 1.32 ^d^	16.20 ± 1.42 ^b^
PO	93.14 ± 0.29 ^c^	55.10 ± 0.33 ^e^	57.31 ± 0.22 ^c^	65.63 ± 0.84 ^d^	50.56 ± 1.00 ^cd^	3.22 ± 0.00 ^a^
PT	25.05 ± 1.31 ^k^	-	7.21 ± 0.23 ^l^	-	19.09 ± 0.44 ^d^	ND
SX	49.99 ± 0.67 ^h^	-	18.07 ± 1.01 ^h^	-	58.16 ± 1.28 ^cd^	212.32 ± 2.08 ^d^
Acarbose	-	-	-	-	-	211.19 ± 1.70 ^d^
Trolox	89.71 ± 0.28 ^d^	8.26 ± 0.11 ^a^	95.90 ± 0.00 ^a^	7.00 ± 0.02 ^a^	4649.69 ± 61.54 ^a^	-

^a–n^ Different superscript letters within each column indicate significant differences (Tukey’s HSD, *p* ≥ 0.05, n = 3). The ABTS and DPPH inhibitions were assayed at a final concentration of 50 µg/mL, and the extract with inhibition higher than 50% was selected to calculate an IC_50_ value. ND means not detected.

**Table 4:** Selection for hub targets using degree, betweenness, and closeness methods with CytoHubba-plugin.

Rank	Degree	Betweenness	Closeness
1	AKT1	ALB	AKT1
2	CTNNB1	ESR1	CTNNB1
3	EGFR	CTNNB1	ESR1
4	STAT3	AKT1	EGFR
5	SRC	EGFR	STAT3
6	ESR1	HMGCR	SRC
7	JUN	TNF	JUN
8	TNF	PTGS2	TNF
9	CCND1	STAT3	CCND1
10	EP300	CYP3A4	MAPK1

**Table 5:** Target genes in signaling pathways related to polyphenols in 15 northeastern Thai local edible plant extracts and T2DM.

KEGG ID	Signaling Pathway		False Discovery Rate (FDR)	Target Genes
hsa04931	Insulin resistance pathway	1.42 × 10^−27^	CPT1B, CREB1, AKT1, AKT2, G6PC1, GSK3B, INSR, NOS3, PIK3CA, PIK3CB, PIK3R1, PIK3CB, MAPK8, MAPK10, PTPN1, PYGL, RELA, STAT3, TNF	
hsa04933	AGE-RAGE signaling pathway in diabetic complications		3.40 × 10^−30^	CDK4, AKT1, AKT2, F3, ICAM1, JUN, SMAD3, MMP2, NOS3, NOX4, SERPINE1, PIK3CA, PIK3CB, PIK3R1, PRKCA, MAPK1, MAPK8, MAPK10, CCND1, RELA, STAT1, STAT3, TNF, VEGFA

**Table 6:** List of 15 northeastern Thai local edible plant samples used for extraction of phytochemical compounds.

Code	Botanical Name	Common Name (Thai Name)	Family	Plant Part	Voucher Specimen No.
AP	*Adenanthera pavonine* L.	Red lucky seed (Maklam)	Fabaceae	Leaves	MSU.PH-FAB-AP01
AV	*Amaranthus viridis* L.	Green amaranth (Khom)	Amaranthaceae	Leaves	MSU.PH-AMA-AV01
BA	*Basella alba* L.	Malabar spinach (Plang)	Basellaceae	Leaves	MSU.PH-BAS-MS01
BR	*Brassica rapa* L.	Chinese cabbage (Kat)	Brassicaceae	Leaves	MSU.PH-BRA-BR01
CaA	*Careya arborea* Roxb.	Wild guava (Kra Don)	Lecythidaceae	Leaves	MSU.PH-LEC-CA01
CeA	*Centella asiatica* (L.) Urban	Asiatic pennywort (Buabok)	Apiaceae	Leaves	MSU.PH-API-CA01
CI	*Clerodendrum infortunatum* L.	Hill glory bower (Khi Khom)	Lamiaceae	Leaves	MSU.PH-LAM-CI01
HC	*Houttuynia cordata* Thunb.	Fish mist (Plu Khao)	Saururaceae	Leaves	MSU.PH-SAU-HC01
LF	*Limnocharis flava* (L.) Buchenau	Yellow velvetleaf (Kan Chong)	Alismataceae	Leaves	MSU.PH-ALI-LF01
LA	*Limnophila aromatica* (Lam.) Merr.	Rice paddy herb (Kra Yaeng)	Plantaginaceae	Leaves	MSU.PH-PLA-LA01
MaC	*Marsilea crenata* C. Presl	Water clover (Waen)	Marsileaceae	Leaves	MSU.PH-MAR-MC01
MoC	*Momordica charantia* (L.)	Bitter melon (Mara Khi Nok)	Cucurbitaceae	Leaves	MSU.PH-CUC-MC02
PO	*Persicaria odorata* (Lour.) Soják	Vietnamese coriander (Phaeo)	Polygonaceae	Leaves	MSU.PH-POL-PO01
PT	*Psophocarpus tetragonolobus* (L.) D.C.	Winged bean (Thua Phu)	Fabaceae	Fruits	MSU.PH-FAB-PT01
SX	*Solanum xanthocarpum* Schrad. & Wendl.	Thai eggplant (MakhueaPro)	Solanaceae	Fruits	MSU.PH-SOL-SX01

## Data Availability

The original contributions presented in this study are included in the article. Further inquiries can be directed to the corresponding author.
